# Research Updates for Influenza Virus and Vaccine Development

**DOI:** 10.3390/vaccines9040383

**Published:** 2021-04-14

**Authors:** Ewan P. Plant, Hang Xie

**Affiliations:** Division of Viral Products, Center for Biologics Evaluation and Research, US Food and Drug Administration, Silver Spring, MD 20993, USA

## Introduction

While the scientific community has been focusing on combating novel severe acute respiratory syndrome coronavirus 2 (SARS-CoV-2) that is responsible for the current COVID-19 pandemic, we also want to draw your attention to this Special Issue of *Vaccines* entitled “Influenza Virus and Vaccine Development”. This Special Issue collects one review and seven research papers that cover multiple aspects of influenza vaccine development, including the development of candidate vaccine virus (CVV) and master donor viruses for generating high growth reassortants, new vaccines and adjuvants, assay evaluation and vaccine safety. These investigations provide a wealth of experience and lessons from recent influenza pandemics, which can also be applied to current COVID-19 vaccine development.

The review article by Rockman, Laurie and Barr [1], highlights lessons learned from the 2009 pandemic and describes how pandemic vaccine capacity, speed of manufacture, and types of vaccine have improved. They note some of the remaining challenges including the Nagoya Protocol, surveillance, and immunogenicity in the elderly. They also express the need to generate CVV strains specific for cell-based vaccine manufacture platforms. The paper by Chen et al. [2] describes the development of master donor viruses for the generation of high growth reassortants to improve the yield of avian H5 and H7 vaccines. In addition to HA yield, Chen et al., also demonstrated that the Vero-cell derived antigens could elicit a robust hemagglutination inhibition (HI) antibody response in mice.

Four other manuscripts in this Special Issue report how novel vaccines can increase the strength and breadth of immune responses beyond the traditional HA response in animal or clinical studies. Bazhan et al. [3] describe a DNA vaccine that combines epitopes from the highly conserved M2 influenza protein with H1 and H3 epitopes. After intramuscular injection, immunized mice had a humoral response and better survival against lethal challenge using either H1N1 or H3N2 viruses. The paper by Tannig et al. [4] describes the use of genetic adjuvants to modulate the immune response in mice administered a DNA vaccine and promote HA-specific IgG isotype production. In the third paper, Ito et al. [5] adapted a live attenuated measles vaccine—licensed in Japan—as the backbone to express influenza H1HA. The resultant H1HA bearing live attenuated measles vaccine is able to elicit both humoral and cellular immune responses in cotton rats. Vaccinated cotton rats survived virus challenge with reduced viral titers and lung damage compared to controls. The research article by Kiseleva et al. [6] provides data from a phase I double-blind, randomized, placebo-controlled clinical trial that investigated a new H7N9 live attenuated influenza vaccine with antigenicity similar to recent viruses detected in humans. Shedding virus was recovered from subjects and was shown to retain the phenotypic and genotypic features of an attenuated virus. Immune responses were also assessed and Kiseleva et al. discuss these in the context of other pandemic vaccine trials. These studies demonstrate that novel vaccines have the potential to improve the breadth of the immune response.

In this Special Issue, Carnell et al. [7] focused on the HI assay specific for influenza B viruses. The HI assay measures the ability of antibodies to prevent viruses from agglutinating red blood cells and an HI antibody titer of 40 is considered the seroprotective threshold after vaccination. Carnell et al. compared the HI assay with other assays for the evaluation of influenza B-specific antibody titers and assessed the lineage-dependent correlation between assays.

The paper by Ambati et al. [8] investigated the potential cause of narcolepsy—a sleep disorder that has been associated with influenza infections and vaccinations. Ambati et al. used mass spectrometry to characterize vaccine lots connected with different incidences of narcolepsy after vaccination. Their results show that the differing composition of influenza proteins in the lots and the mutational burden of the virus seeds used to generate the vaccines might be contributing factors in narcolepsy susceptibility.

We sincerely hope these lessons learned from past and current influenza research will benefit the ongoing battle against the COVID-19 pandemic.

## Data Availability

Not applicable.
